# *Neotermescostaseca*: a new termite from the coastal desert of Peru and the redescription of *N.chilensis* (Isoptera, Kalotermitidae)

**DOI:** 10.3897/zookeys.811.30809

**Published:** 2018-12-31

**Authors:** Rudolf H. Scheffrahn

**Affiliations:** 1 Fort Lauderdale Research and Education Center, University of Florida, 3205 College Avenue Davie, Florida 33314, USA University of Florida Davie United States of America

**Keywords:** New species, Neotropics, Chile, imago, soldier

## Abstract

The imago and soldier castes of a new *Neotermes* species, *N.costaseca*, are described. It is only the third termite species known from the Pacific coastal desert of Peru. *Neotermescostaseca***sp. n.** is compared with the allopatric *Neotermeschilensis* from the arid central and southern coastal plain of Chile.

## Introduction

The coastal desert of Peru and Chile (Atacama) spans approximately 3,000 km from -5° to -27° latitude. Only two termite species are recorded from this region, *Cryptotermesbrevis* (Walker) (widespread, Scheffrahn et al. 2009) and *Amitermeslunae* in the north (Trujillo, Peru; Scheffrahn and Huchet 2010). Another species in the genus *Neotermes* Holmgren, 1911, *N.chilensis* (Blanchard), extends northward to the steppe transition zone of the Atacama (Copiapo, Chile) and ranges southward along the coastal plain to Santa Cruz, Chile (Camousseight and Vera 2005). Herein, a new *Neotermes* species is described, *N.costaseca*, the third species of termite from the Peruvian coastal desert, and it is compared with the Chilean *N.chilensis*.

## Materials and methods

Microphotographs were taken as multi-layer montages using a Leica M205C stereomicroscope controlled by Leica Application Suite version 3 software. Preserved specimens were taken from 85% ethanol and suspended in a pool of Purell® Hand Sanitizer to position the specimens on a transparent Petri dish background.

## Taxonomy

### Neotermes costaseca sp. n.

Taxon classificationAnimaliaBlattodeaKalotermitidae

http://zoobank.org/94D33072-E4B6-4251-96DC-CF1DB7D9E429

**Figure 1.**
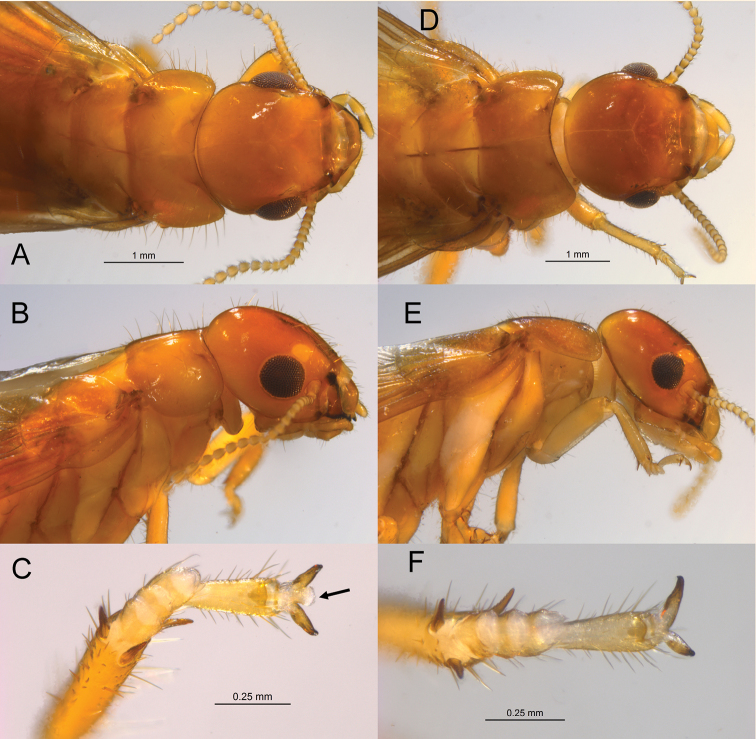
Dorsal and lateral views of head and pronotum and fore tarsi of *Neotermes* alates. **A–C***N.ostaseca* (arrow = arolium) **D–F***N.chilensis*.

**Figure 2.**
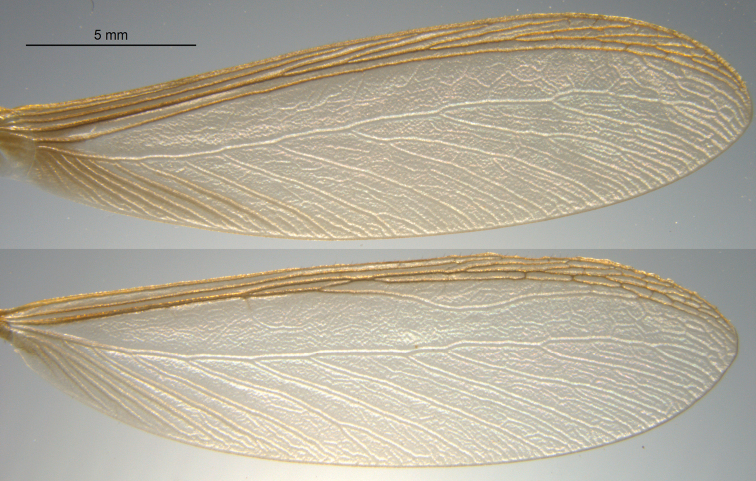
Fore and hind wing of the *Neotermescostaseca* alate.

**Figure 3.**
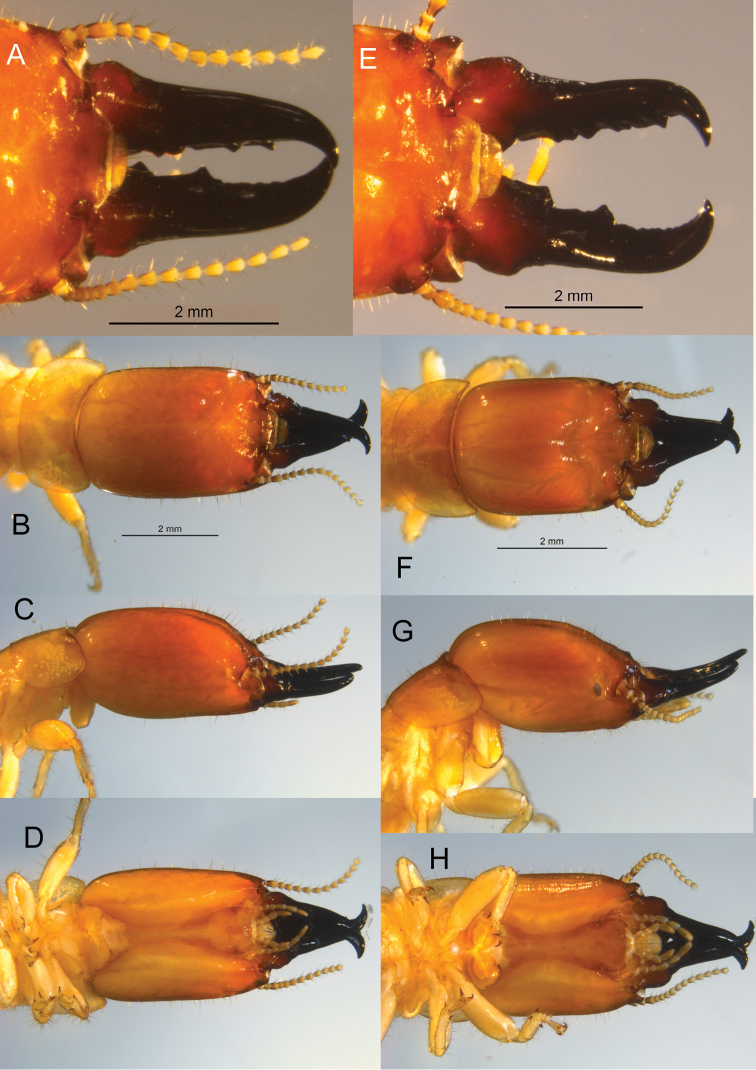
Open mandible, dorsal, lateral, and ventral views of soldier head capsule and pronotum of *Neotermes* soldiers. **A–D***Neotermescostaseca* and **E–H***N.chilensis*.

**Figure 4.**
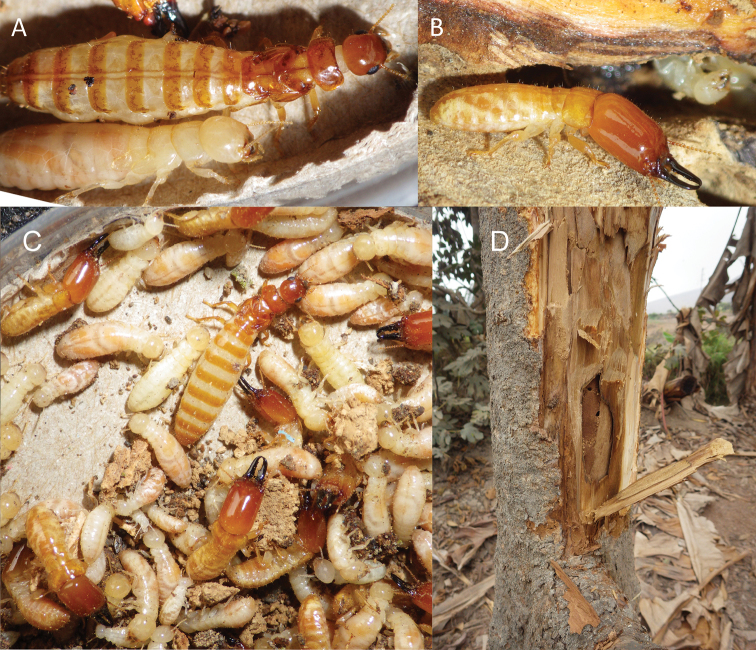
Live habitus photographs of *Neotermescostaseca*; **A** physogastric queen and pseudergate, colony 1 **B** soldier, colony 1 **C** various castes, colony 2 **D** exposed galleries of live tree from where colony 2 was removed.

Figures 1A–C , 2 , 3A–D , 4 ; Tables 1 , 2

#### Diagnosis.

The imago of *N.costaseca* has larger eyes and ocelli than *N.chilensis* and the former possesses arolia. The soldier mandible of *N.chilensis* has much more pronounced basal humps than *N.costaseca* and the former has more protruding genal horns.

#### Description.

*Imago* (Figs 1A–C, 2; Table 1). Head capsule and pronotum orange-brown. Compound eye nearly circular; ocellus yellowish orange, large, and roundly ellipsoid; nearly touching eye margin. Head vertex and frons slightly depressed, without rugosity; covered with dozens of long (0.25 mm), variously directed setae. Pronotum wider than head capsule; anterior margin evenly concave; anterior margin very weakly emarginate. Pronotum covered with shorter and many long (0.3 mm) setae, especially along lateral margins. Antennae with 17–22 articles, basal article relative lengths 1>2=3>4. Anterior margin of fore wing scale convex; margin lined with 12–15 setae. Fore wing with subcosta joining costal margin at ca. one-fifth of wing length from suture. Radius joining costal margin at two-fifths wing length; radial sector with ca. seven anterior branches. Median vein sclerotised and running very close to and parallel radial sector. Arolium present.

**Table 1.** Measurements (mm) of *Neotermes* imagos’ characters.

**Characters**	**Females, 3 colonies (n = 8)**		**Males, 5 colonies (n = 10)**	
***Neotermescostaseca*, sp. n.**	**mean**	**range**	**mean**	**range**
Head width, maximum (w/out eyes)	1.7	1.60–1.90	1.69	1.66–1.72
Head width, maximum (with eyes)	1.84	1.76–1.96	1.85	1.78–1.94
Pronotum, maximum width	2.07	1.98–2.13	2.02	1.97–20.9
Eye diameter, maximum	0.56	0.50–0.60	0.57	0.50–0.61
Body length	8.56	7.14–13.17	8.47	6.80–9.40
Right forewing length	14.67	14.00–16.35	15.41	13.00–16.19
Body length with wings	17.43	16.51–17.94	17.59	16.30–18.73
Number of antennal articles	18.9	17–21	19.75	17–22
*** Neotermes chilensis ***	**Females, 1 colony (n = 1)**		**Males, 2 colonies (n = 2)**	
	**mean**	**range**	**mean**	**range**
Head width, maximum (w/out eyes)	1.74	1.70–1.78	1.64	1.63–1.66
Head width, maximum (with eyes)	1.82	1.82–1.82	1.79	1.78–1.80
Pronotum, maximum width	2.04	1.95–2.13	1.97	1.91–2.03
Eye diameter, maximum	0.47	0.44–0.51	0.46	0.44–0.47
Body length	8.57	6.51–10.63	7.2	6.40–8.00

*Soldier* (Fig. 3A–D, Table 2). Head capsule in dorsal and lateral aspect orange-brown; ventrally yellowish orange; pronotum yellowish orange. Eye spot yellow; small, narrow ellipsoid. Head and pronotum covered with shorter and moderately long (0.15–0.25 mm) setae; seta denser and longer on frontal lobes. Head capsule with lateral margins nearly parallel, slightly converging at anterior; genal horns not protruding in ventral view. Frons roundly sloping 45° from vertex; depressed and slightly rugose from width of postclypeus to anterior vertex. Y-suture distinct, narrow. Pronotum width 2.5× length, posterior margin slightly concave, posterolateral corners rounded 90°. Antennae with 14–16 articles, basal article relative lengths 1>2<3>4. Mandibles with shallow basal hump more than 4/5^th^ length from apical points. Mandibles evenly curved ca. 80° along apical third.

**Table 2.** Measurements (mm) of *Neotermes* soldier characters.

Characters	*Neotermescostaseca* (n = 10)		*Neotermeschilensis* (n = 10)	
	mean	range	mean	range
Head length to lateral mandible base	3.46	2.66–3.92	3.25	2.80–3.84
Head width, maximum	2.44	2.25–2.66	2.63	2.31–2.97
Head height with gula, maximum	2.04	1.72–2.30	1.94	1.75–2.13
Pronotum length	1.38	1.20–1.69	1.54	1.25–1.84
Pronotum width	2.60	2.38–3.06	2.74	2.38–3.19
Number of antennal articles	15	14–16	16.22	14–18
3^rd^ antennal article length	0.17	0.14–0.19	0.23	0.19–0.28

**Holotype**: Perú: Lurin, Rio Lurin, (-12.275, -76.879), 23OCT2007, J Křeček (JK); labelled soldier (University of Florida Termite collection no. PE131).

#### Material examined.

Perú: Lurin, Rio Lurin (-12.275, -76.879), 23OCT2007, J. Křeček (JK); winged imagos, soldiers, pseudergates (UF no. PE131). Perú: Lima, Rio Chillon (-11.979, -77.090), 20OCT2007, JK; winged imagos (UF no. PE107). Perú: Lima, Rio Lurin, Quebrada Verde bridge (-12.237, -76.856), 23OCT2007, JK, Gerardo; winged imagos (UF no. PE117). Perú: Lima, Rio Lurin, Quebrada Verde bridge (-12.237, -76.856), 23OCT2007, JK, Gerardo; winged imagos (UF no. PE119). Perú: Lima, Huaral (-12.275, -76.879), 23OCT2007, JK, Gerardo; winged imagos (UF no. PE131). Perú: Lima, Rio Lurin (-11.521, -77.239), 25OCT2007, JK, C. Torres; winged imagos (UF no. PE145). Perú: Lima, Chacra y Mar (-11.60804, -77.23939), 25OCT2007, T. Carrijo R. Constantino, J. Chase, J. Křeček, E. Kuswanto, J. Mangold, A. Mullins, T. Nishimura, R. Scheffrahn (CCCKKMMNS); winged imagos (UF no. PU1012). Perú: Ancash, Huaylas (-8.872, -77.894), 9MAY2014, CCCKKMMNS; soldiers (UF no. PU1019). Perú: Parque Nat. Lachay (-11.363, -77.371), 9MAY2014, CCCKKMMNS; soldiers (UF no. PU1007). Perú: Lima, Chacra y Mar (-11.608, -77.239), 3JUN14, CCCKKMMNS; soldiers (UF no. PU1011). Perú: Lima, Huaral, Pueblo Libre, Rio Chancay bridge (-11.514, -77.230), 23OCT2007, JK, C. Torres; soldiers (UF no. PE135). Perú: Lima, Rio Lurin, Quebrada Verde bridge (-12.237, -76.856), 23OCT2007, JK, Gerardo Torres; soldiers (UF no. PE120). Perú: Lima, Pachacamac (-12.243, -76.864), 23OCT2007, JK, Gerardo; soldiers (UF no. PE126). *Neotermesfulvescens*, Paraguay: Dry Chaco Mariscal Estigarribia (-22.078, -60.552), 1JUN2012, J. Chase; soldiers and queen (UF no. PA742).

#### Etymology.

From Spanish, meaning “dry coast” and describing the species’ habitat; to be treated as a noun in apposition.

#### Comparisons.

Although climatically isolated, some character overlap is possible with other neotropical *Neotermes*. A revision of Neotropical *Neotermes* is needed to identify diagnostic characters. The imago of *N.costaseca* has longer head and pronotal setae and larger eyes and ocelli than *N.chilensis. Neotermescostaseca* has an arolium which is lacking in *N.chilensis*. The soldier mandible of *N.chilensis* has much more pronounced basal humps, more sinuate marginal teeth, and more sharply curved apical teeth than *N.costaseca*. The anterolateral corners of *N.chilensis* constrict more than those of *N.costaseca* and, unlike *N.costaseca*, the genal horns of *N.chilensis* protrude. The pronotum of the *N.chilensis* soldier is crescent-shaped with that of *N.costaseca* resembles a bow tie. The soldier eye spot of *N.costaseca* is hyaline while that of *N.chilensis* is almost always pigmented. The soldier of *N.castaneus* differs from both *N.costaseca* and *N.chilensis* in having shorter, thicker mandibles with larger, more rounded basal humps.

The arid-adapted *N.costaseca* and *N.chilensis* are most comparable with non-Amazonian congenerics from Argentina (Torales et al. 2008), Bolivia, Paraguay, and southern Brazil (Krishna et al. 2013). Compared to *N.chilensis*, the soldiers of *N.hirtellus* (Silvestri), *N.fulvescens* (Silvestri), and *N.modestus* (Silvestri) all have more reduced madibular basal humps (Silvestri 1903). Compared to *N.costaseca* and *N.chilensis*, the *N.hirtellus* soldier has a shorter third antennal article relative to the second, the head converging toward the front, and the ocellus separated from eye (Costa Lima 1941). The imago and soldier of *N.fulvescens* are smaller, the imago lacks an arolium, and the solder mandibles are shorter and thicker than both *N.chilensis* and *N.costaseca*. The *N.modestus* soldier and imago are smaller than both *N.chilensis* and *N.costaseca* (Silvestri 1903). Compared to *N.chilensis* and *N.costaseca*, the imago wings of *N.arthurimuelleri* (Rosen) are more darkly pigmented, shorter (12 mm long), and the ocelli are more separated from the eye (Costa Lima 1942).

The *N.glabriusculus* Oliveira imago has smaller ocelli than both *N.costaseca* and *N.chilensis* and are more removed from the eye while the soldier dentition in the former is less robust and the tips are not as curved and have almost no basal humps (Oliveira 1979). The *N.magnoculus* (Snyder) imago is smaller (Snyder 1926) than *N.chilesnsis* and N.costaseca. The *N.wagneri* (Desneux) soldier has proportionally shorter mandibles and no enlargement of the third antennal article compared with *N.chilesnsis* and *N.costaseca* (Costa Lima 1941, 1942). Finally, the *N.zanclus* Oliveira soldier has a more elongated and sub rectangular head capsule (Oliveira 1979).

#### Biology.

*Neotermescostaseca* colonies were collected from both dead branches attached to live trees and directly from sapwood within live trees (Fig. 4). Alates were present in October, but were not collected in June suggesting the latter as part of the likely flight season. The greater tergite separation and mottling in the queen depicted in Fig. 4A suggest that this queen is older than the queen in Fig. 4C

### Neotermes chilensis

Taxon classificationAnimaliaBlattodeaKalotermitidae

(Blanchard, 1851)

Figures 1D–F , 3E–3 , 4 ; Table 1 , 2

#### Synonyms.

See Krishna et al. 2013: 538–539 for complete synonymic list. Camousseight and Alehandro 2005: 39–45, synonymy; measurements; soldier, worker mandibles figured. Ripa and Luppichini 2004: 69–71, Chile termite key; 84, alate and soldier illustrated and photographed.

#### Description.

*Imago* (Fig. 1D–F; Table 1). Head capsule and pronotum reddish brown. Anterodorsal margin of compound eye straight; ocellus yellowish orange, reniform; touching eye margin. Head vertex and frons slightly depressed, slightly rugose; covered with scattered short setae (0.15–0.2 mm) directed forward on head, variably directed on pronotum. Pronotum wider than head capsule; anterior margin evenly concave; anterior margin emarginate giving “bow tie” resemblance. Pronotum covered with short and medium-long (0.15–0.25 mm) setae. Anterior margin of fore wing scale convex; margin lined with 15–20 setae of same length. Arolium absent.

*Soldier* (Fig. 3E–H, Table 2). Head capsule in dorsal and lateral aspect orange-brown; ventrally lighter; pronotum yellowish orange. Eye spot dark; small, ellipsoid. Head and pronotum covered with short (0.1–0.15 mm) setae; seta more dense and longer on frontal lobes. Head capsule with lateral margins parallel, converging to ~ 85% of width at anterior; genal horns protruding in ventral view. Frons sloping gradually ~ 30° from vertex; depressed from width of postclypeus to middle of vertex. Pronotum crescent-shaped, posterior margin evenly rounded to the anterolateral corners. Antennae with 14–18 articles, basal article relative lengths 1>2<3>4. Mandibles with robust basal hump more ~ 3/4^th^ length from apical points. Mandibles abruptly curved ca. 90° along apical fourth. Dentition robust, undulating.

#### Material examined.

Chile: La Serena, Road 5, Ovalle-Quebrada Seca intersection (-27.356, -70.659), 6OCT2007, JK, R. Ripa, P. Luppichini; imago (UF no. CL26). Chile: Atacama, 3km E PN Llanos de Challe (-30.518, -71.484), 5OCT2007, JK, R. Ripa, P. Luppichini; soldier (UF no. CL21). Chile: La Serena, PN Borque Fray Jorge (-30.667, -71.675), 6OCT2007, JK, R. Ripa, P. Luppichini; soldier (UF no. CL30). Chile: Valparaiso, La Cruz (-32.852, -71.183), 9OCT2007, JK, R. Ripa, P. Luppichini; soldier (UF no. CL33). Chile: Valparaiso, La Cruz (-32.852, -71.183), 9OCT2007, JK, R. Ripa, P. Luppichini; soldier (UF no. CL34). Chile: Santiago de Chile, Mallarauco (-33.459, -70.635), 11MAR1997, M. Rust; soldier (UF no. CL49). Chile: Santiago de Chile (-33.459, -70.635), 15FEB1999, J. Hughes; imago (UF no. SA158). Syntypes deposited in the Muséum National d’Histoire Naturelle, Paris, were unavailable and not examined.

#### Biology.

*Neotermeschilensis* colonies were collected from fence posts, dead branches, and dead tree trunks. An alate was collected in mid-February suggesting a late summer flight season.

#### Discussion.

**Figure 5.**
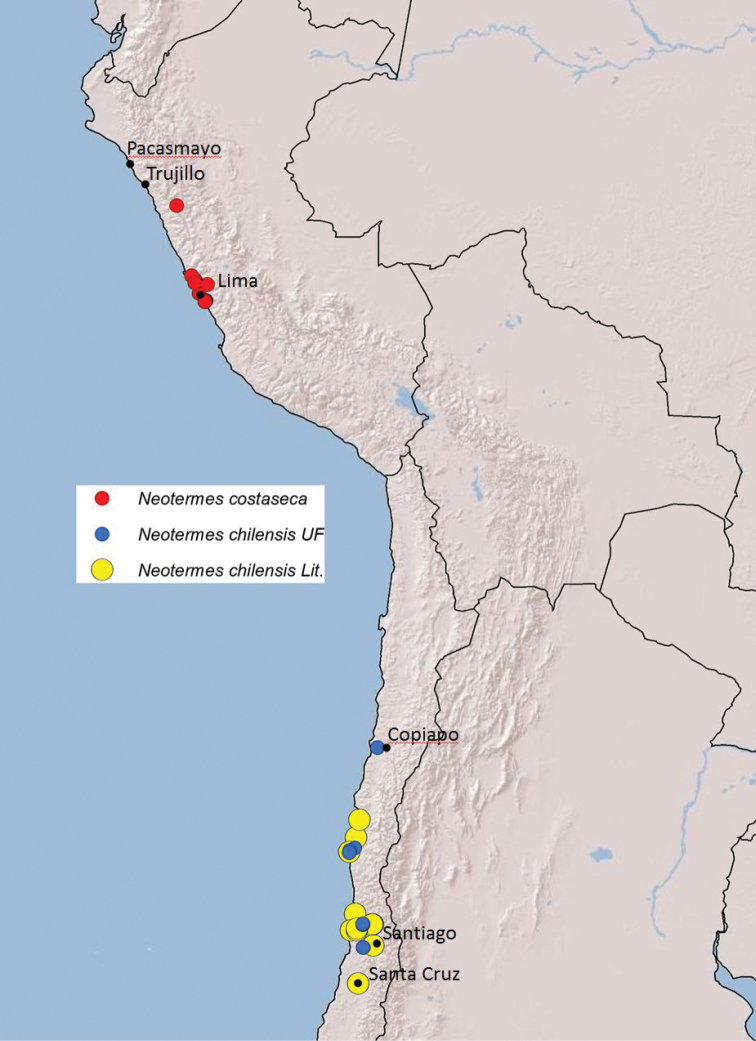
Map of *Neotermescostaseca* and *N.chilensis* localities.

The lack of termite diversity in the Neotropical coastal desert can be attributed to its climate and geographical barriers of the Pacific Ocean and the Andes. The entire coast of Peru and much of the Chilean coast is arid, but profound aridity (≤ 20 mm/yr) begins near Pacasmayo, Peru, and extends southward to approximately Copiapo, Chile (Fig. 5, climate data from http://www.weatherbase.com/). Although hyperarid as a result of Humboldt Current cooling, this region is transected by many wooded riparian habitats fed by rain and snowmelt runoff from the Andes, providing food (wood) for only three termite species.

With the addition of *N.costaseca*, there are now 27 *Neotermes* species (Krishna et al. 2013). With only *N.costaseca* and two other termite species known from the coastal desert of Peru and Chile, it is unlikely that *N.costaseca* will be found outside of this unique Neotropical biome.

## Supplementary Material

XML Treatment for Neotermes costaseca

XML Treatment for Neotermes chilensis
